# Accounting for Visual Field Abnormalities when Using Eye-tracking to Diagnose Reading Problems in Neurological Degeneration

**DOI:** 10.16910/jemr.17.2.2

**Published:** 2024-07-04

**Authors:** Carla D. Guantay, Laura Mena-García, Miguel Ángel Tola-Arribas, María José Garea García-Malvar, Marta Para-Prieto, Gloria González Fernández, Agustín Mayo-Iscar, J. Carlos Pastor

**Affiliations:** Instituto Universitario de Oftalmobiología Aplicada (IOBA Eye Institute), Universidad de Valladolid, Valladolid, Spain; Red de Investigación Cooperativa Orientada a Resultados en Salud (RICORS), Red de Enfermedades Inflamatorias (REI), Instituto de Salud Carlos III, Madrid, Spain; Department of Neurology, Hospital Universitario Río Hortega, Valladolid, Spain; Centro de Investigación Biomédica en Red en Bioingeniería, Biomateriales y Nanomedicina (CIBER-BBN), Madrid, Spain; Department of Ophthalmology, Hospital Clínico Universitario, Valladolid, Spain; Department of Statistics and Operational Research and IMUVa, Universidad de Valladolid, Valladolid, Spain

**Keywords:** eye movement, eye tracking, saccades, area of interest, gaze, reading, neurological degeneration

## Abstract

State-of-the-art eye trackers provide valuable information for diagnosing reading problems by measuring and interpreting people’s gaze paths as they read through text. Abnormal conditions such as visual field defects, however, can seriously confound most of today’s existing methods for interpreting reading gaze patterns.

Our objective was to research how visual field defects impact reading gaze path patterns, so the effects of such neurological pathologies can be explicitly incorporated into more comprehensive reading diagnosis methodologies. A cross-sectional, non-randomized, pilot clinical study including 45 patients with various neurologic disorders and 30 normal controls was designed. Participants underwent ophthalmologic/neuropsychologic and eye-tracker examinations using two reading tests of words and numbers.

The results showed that the use of the eye tracker showed that patients with brain damage and an altered visual field require more time to complete a reading-text test by fixating a greater number of times (p < 0.001); with longer fixations (p = 0.03); and a greater number of saccades in these patients (p = 0.04). Our study showed objective differences in eye movement characteristics in patients with neurological diseases and an altered visual field who complained of reading difficulties. These findings should be considered as a bias factor and deserve further investigation.

## Introduction

Oculomotor abnormalities may be consequences of many neurologic diseases (37). However systematic examination of the oculomotor systems in patients with neurologic disorders is not currently a routine clinical practice. Nevertheless, there are patients who do not show clear oculomotor alterations, but they complain of reading difficulties (29).

On the other hand, in many brain-damaged patients these reading disorders may be caused by visual field defects (VFD) (34).

Previous research has shown that analysis of the reading process in these patients is very important because it provides information about perceptual preconditions and cortical adaptive strategies, which are also important for rehabilitation purposes (34).

For reading assessment, it is easy to count the number of words a patient can read per minute, but characterizing the reading pattern requires more complex explorations. Nevertheless, as mentioned, neurologists and ophthalmologists do not use these techniques routinely (13).

Some authors have postulated that a careful examination of eye movements and the evaluation of vestibular function, supplemented with new objective recording techniques to quantify the findings, should be part of the standard neurologic examination protocol (17).

Objective recordings of eye movements through eye-tracking systems are not currently performed widely, despite that they have been described as an important adjunct in diagnosis, documentation, and management of different neurologic conditions (7).

Various recording systems have been implemented over the years, such as OSCANN (Aura Innovative Robotics SL, Madrid, Spain), a video-oculography system, has been used to analyze Alzheimer's disease, Parkinson's disease, cirrhosis, type II diabetes, minimal hepatic encephalopathy, and epilepsy.

One of the most important advantages of these technologies is their relative ease of handling and non-invasiveness (15; 17). However, they have some limitations, for example in capturing some special eye movements, such as microsaccades (15).

Eye tracking systems are another option. They capture eye movements with video cameras using the reflection of infrared light (provided by infrared emitting light-emitting diodes) over the corneal limbus and the central point of retinal reflection through the pupillary aperture (7).

During the reading assessment, the eye tracker allows different types of eye movements to be distinguished with great precision, especially fixations. Fixations are the pauses during which the eyes are relatively still in order to extract and process information from a small section of text. Saccadic movements correspond to small jumps that the eyes make in the right direction horizontally (left to right) from one group of letters to another (19; 20; 30). In addition, anti-saccadic movements occur when the eyes move in the opposite direction (from right to left) to review a piece of text that the patient has difficulty to understand.

Most patients with acquired brain diseases experience reading problems, although as mentioned, a systematic evaluation of the reading capability and pattern of those patients is not incorporated in most of the medical protocols for managing them. Patients are asked about diplopia or reading difficulties only in a few cases (36).

Multiple sclerosis (MS) is the leading cause of non-traumatic disability in young adults. During the disease course, oculomotor abnormalities may be present in up to 95% of patients (11). By using video-oculography, Polet et al. confirmed that oculomotor abnormalities are common in all MS phenotypes, even in the early stages, suggesting that this technology can be useful for detecting the demyelinating process in the preclinical phase, highlighting these subclinical abnormalities even in the absence of characteristic lesions visible on magnetic resonance imaging (28; 39).

The irregularities described in these patients can be characterized by inaccurate fixations, prolonged saccadic latency, reduced saccade speed, and symptoms associated with visual fatigue (25; 37).The commonest reported abnormal ocular movements in advanced MS and clinically isolated syndrome was saccadic dysmetria (41.7%) and altered smooth tracking (42.3%) (21; 38).

The presence of oculomotor disorders also was reported to be correlated with a higher level of disability and is generally linked to a worse prognosis (1).

Cerebral vascular diseases are a frequent cause of morbidity and hospitalization in the population and involve a very high social health expense (5). Previous studies have shown that the incidence and prevalence of visual problems in patients with strokes affect more than half of the survivors (29). Among them, VFD occurs in from 20% to 57% of cases and they could impact mobility in daily life, driving and reading, among other visual functions (4; 36).

Parkinson's disease is another frequent neurodegenerative disease associated with alterations of oculomotor problems and reading difficulties (40). Among patients with Parkinson's disease, 78% reported at least one visual symptom, including difficulties in reading (2). Reading tests have shown a decrease in the number of words read per minute, but few papers deal with the reading patterns (41). Additionally, oculomotor disorders include altered saccadic oculomotor performance, greater numbers of saccades and interruptions with long regressive movements, stepped saccadic movements, and increased latency and saccadic hypometria (22; 45). However, their analysis has not been incorporated into the routine examination of those patients.

In brief, eye trackers results are being used progressively as a biomarker for neurodegenerative diseases but they can be affected by the presence of VFD thus introducing a bias to evaluate the possible specific alteration of motor control systems.

The relationship between VFD and ocular movement abnormalities is well known. For example, it is already known that hemianopsia leads to a malfunction of microsaccade control circuits that worsens over time (12).

In summary, eye tracking systems are progressively being used as biomarkers of neurodegenerative diseases, but their recordings can be affected by the presence of alterations in VF, which could induce a bias in the interpretation of reading patterns, an influence that has not been previously published.

Thus, the objective of the current research was to find out what is the influence of VFD in the reading pattern analyzed by the eye tracking device, Tobii Pro Nano Hardware Package® (Tobii Pro AB, Danderyd, Sweden) in patients with neurological pathologies complaining with reading difficulties.

## Methods

### Participants and Design

An independent ethical review board approved this research (code PI 21-2247 TFM), which conforms to the principles and applicable guidelines for the protection of human subjects in biomedical research. All procedures were performed in accordance with the Declaration of Helsinki. All candidates provided written informed consent. The study was registered in Clinical Trials (NCT04937725).

This cross-sectional, non-randomized, pilot clinical study of cases and controls included 45 patients with acquired brain diseases and 30 controls recruited from 3 clinical centers. The main inclusion criteria for patients were age between 18 and 80 years, complaining of reading difficulties, clinical neurologic and radiologic stability, in those with acute pathologies (≥3 months after acute cerebral disease), and no visual hemi-neglect assessed using the Clock Drawing test and the Line Bisection test. Patients with visual agnosia evaluated with the Poppelreuter-Ghent test and cognitive deficits evaluated by the Mini Mental State Examination (MMSE) test were excluded (3). Patients had to have a score of 23 or higher based on MMSE test and sufficient residual hearing ability (3; 23). Other exclusion criteria were participants with a history of maculopathy and advanced cataracts or other ocular diseases that could affect central visual acuity or macular fixation.

### Materials

Ophthalmologic examination comprised evaluation of ocular alignment and motility including observation in primary gaze, cover and uncover tests for far and near distances, and evaluation of extrinsic ocular motility (versions and vergences).

The distance visual acuity was evaluated monocularly and binocularly with an ETDRS chart at 4 meters and the measurements were recorded with their equivalents in decimal scale. Patients with low visual acuity were excluded. Patients with a best corrected visual acuity of 0.5 or greater (decimal scale) and 20/40 (Snellen scale) were included.

For near visual acuity, the Colenbrander optotypes in Spanish (Precision Vision, Woodstock, Illinois, United States of America) were used. The visual acuity was measured binocularly with a printed card at a distance of 40 centimeters; the results were recorded in Snellen scale.

Pupils, anterior pole, and fundus were examined. A 30-2 computerized visual field was performed (Humphrey Perimeter, version 4.2 of the series II system software; Carl Zeiss Meditec, Jena, Germany).

The controls met the same criteria for visual acuity, a normal visual field, and no history of neither neurologic nor ocular diseases that could affect central visual acuity.

Eye movements were recorded with the Tobii Pro Nano Hardware Package® eye-tracking system, associated with an HP OMEN 17-AN025NS Notebook (Hewlett-Packard, Palo Alto, California, United States of America). The Tobii Pro Nano® system is a screen-based eye tracker that captures gaze data at 60 hertz and is designed for fixation-based studies. The results were recorded and analyzed with the Tobii Pro Nano software Package® (Tobii Pro Lab Full Edition Version). With this program, two separate protocols were designed for each word and number reading tests.

### Procedure

During the evaluation, the patient was positioned 50 to 54 centimeters from the screen of a laptop, without any type of head restraint and was instructed to keep his or her head fixed during the calibration of the system and performance of the tests that are described below.

During calibration, the participant had to look at the calibration stimulus on the screen. The stimulus was a circle that moved in 5 target positions and the timed calibration mode offered by the system was used. In this type of calibration, the next calibration point is displayed when the eye-tracker has signaled that it has enough data to continue or when enough time has passed for a reasonable amount of data to be collected.

**Figure 1.**
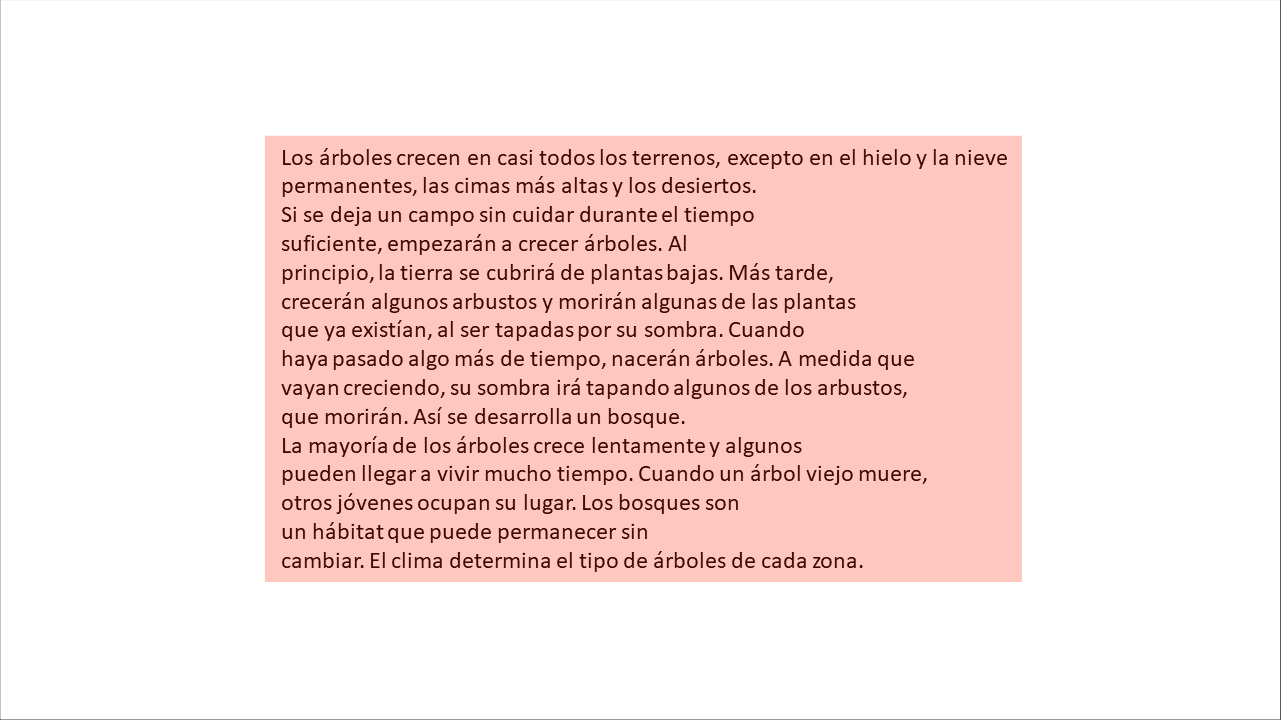
Area of Interest (AoI) analyzed to investigate eye movements using the IReST© test. The AoI has been colored to represent the rectangular area containing the text during the reading test. IReST© = the test used is based on the IReST© test.

The reading assessment was performed using text based on International Reading Speed Texts (IReST©) (Precision Vision, Spanish version, La Salle, Illinois, United States of America) (43). Individuals were instructed to read aloud as fast as possible a selected text from the compilation of adapted with the PowerPoint program, with 14-point Calibri font, with an average length of 141 words (Figure 1). This allowed evaluation of the corrected reading performance in words per minute calculated using the following formula: [(words read − number of errors)/minutes spent reading].

**Figure 2.**
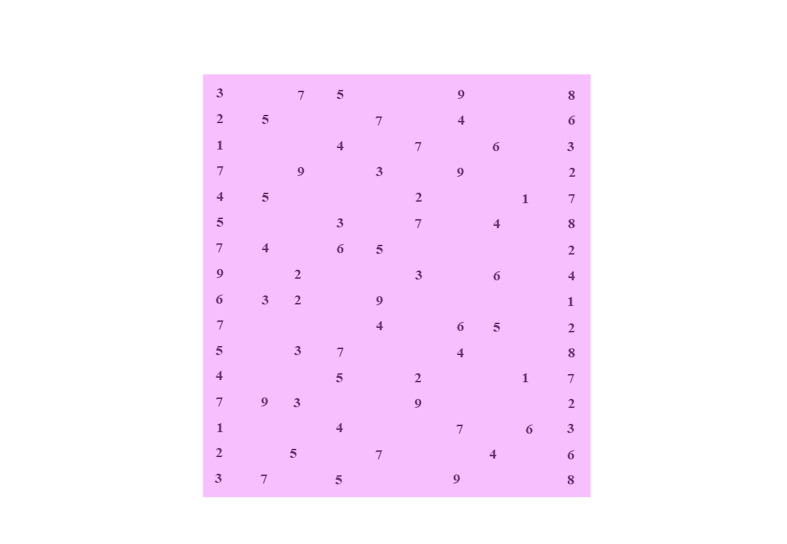
The Area of Interest (AoI) analyzed in the DEM© test. The AoI has been colored to represent the rectangular area containing the series of numbers during the test. DEM© = the test used is based on the DEM© test. DEM© = the test used is based on DEM© test.

The evaluation of the reading of a series of numbers was based on the Developmental Eye Movement test (DEM©) (Bernell Corporation, South Bend, Indiana, United States of America) (10). The DEM© test is comprised of three different plates, but only card C was selected because it evaluates the denomination of numbers in a task similar to horizontal reading. In this examination, the patients were asked to read aloud as quickly as possible a series of numbers adapted with the PowerPoint program in Times New Roman 14-point font (Figure 2). This facilitated evaluation of the corrected reading performance in numbers per minute and was calculated using the following formula: [(numbers read − number of errors)/minutes spent reading].

Both tests were timed manually, and the additions, failures, and omissions were recorded. The data were analyzed considering intervals and areas of interest (AoI) of each protocol. Intervals are periods on the recording timeline that have start and end points. The start point was defined automatically as the moment when the image with the text or the series of numbers appeared on the screen. The end point was defined by the evaluator who pressed a key on the laptop at the moment the patient finished reading aloud the last word or number of the test. The AoI was defined as a rectangle that included the image of each proof. Complete fixation was defined as the one preceded and followed by a saccade; it must be entirely contained in the interval and in the AoI.

Variables selected to study the ocular motility with the eye-tracking system while reading the IReST© test were the duration of the interval expressed in milliseconds, the number of whole fixations in the AoI (rectangle), the total duration of whole fixations in AoI (millisecond), the average duration of whole fixations in AoI (millisecond), the minimal/maximal duration of whole fixations in the AoI (the duration of the shortest and longest fixation expressed in millisecond), the time to first whole fixation in the AoI (millisecond), the duration of the first whole fixation in the AoI (millisecond), and the number of saccades in the AoI.

The whole fixations outside and inside the AoI were measured, and the number, total duration, average duration, and duration of the first fixation recorded.

The variables selected for the evaluation of the reading of the series of numbers were the number of whole fixations in the AoI, the duration of first whole fixation in the AoI (millisecond), and the number of saccades in AoI. In addition, whole fixations inside and outside the AoI were measured: number, total duration, average duration and duration of first fixation.

### Statistical analysis

The data were registered in an Excel database according to the rules established by the Spanish Organic Law 3/2018, December 5, on the Protection of Personal Data. Statistical analyses were performed using the statistical package R, v4.0 (R Foundation for Statistical Computing V, Austria).

Univariate analysis of the variables was initially performed using descriptive statistics using frequency distribution tables for the qualitative variables and measures of means and standard deviation for quantitative variables. The chi-square test was used to identify associations between categorical variables, while Shapiro-Wilk test was used to assess their normality.

Additionally, both the Student’s t-test and the Mann-Whitney test were used to identify relationships between the categorical and numerical variables, considering the level of statistical significance *p* < 0.05.

## Results

The 45 patients (23 men, 22 women) were compared to the 30 controls (19 women, 11 men); the gender difference did not reach significance (p = 0.32). The mean age of the controls was 51 ± 17 years and that of the cases 50 ± 15 years (p = 0.92).

The patients included 28 patients with MS, 7 with Parkinson's disease, 7 with stroke, and 3 with cerebellar syndrome. These subjects were subdivided according to the results of their visual field tests, that is, 33 had a normal visual field (cases with normal visual field = CNVF) and 12 had VFD (cases with affected visual field = CAVF [mean defect, -11.44 decibels]). The VFD included 5 hemianopias, 3 altitudinal defects, 2 centrocecal scotomas, and 2 peripheral defects with central respect.

The 12 CAVF included 5 who had a stroke, 5 suffering MS, and 2 Parkinson's disease. The subgroup of 33 patients with a normal visual field included 23 patients with MS, 5 with Parkinson's disease, 3 with cerebellar ataxia, and 2 with stroke.

The IReST© test did not show significant differences in the words per minute between cases and controls (Table 1). In the cases, a tendency to read fewer words was observed, but the data showed great variability. However, when data were recorded with the eye-tracker system, the duration of interval was longer in patients with an affected visual field compared with the controls (Table 1).

**Table 1.** Comparison between control group and cases in IReST test.

Parameter	Control group (*n*=30)	Cases (*n*=45)	Control (*n*=30) vs. Cases (*n*=45)	CNVF (*n*=33)	Control (*n*=30) vs. CNVF (*n*=33)	CAVF (*n*=12)	Control (*n*=30) vs. CAVF (*n*=12)
IReST	Mean *(SD)*	Mean *(SD)*	*p*	Mean *(SD)*	*p*	Mean *(SD)*	*p*
Words per minute	139.90 (1.52)	137.59 (12.55)	0.78	139.42 (4.57)	0.72	132.56 (23.05)	0.16
Duration of interval (ms)	56012.23 (12369.84)	64664.93 (31068.05)	0.67	61422.00 (32670.12)	0.49	73583.00 (25250.82)	0.01
Number of whole fixations in AoI	129.17 (27.38)	132.36 (43.85)	0.50	122.45 (43.48)	0.59	159.58 (32.99)	<0.001
Total duration of whole fixations	47537.10 (11199.39)	50806.56 (24002.40)	0.92	46422.97 (22209.27)	0.34	62861.42 (25561.09)	0.03
Number of saccades in AoI	112.57 (18.31)	115.33 (41.45)	0.29	110.64 (41.17)	0.75	128.25 (41.14)	0.04

Note. vs.= versus, IReST = the test used is based on IReST*©* test, *SD*= Standard deviation, ms = millisecond, AoI = Area of Interest, CNVF = Cases with Normal Visual Field, CAVF = Cases with Affected Visual Field.

Regarding registration of the fixations, the numbers of whole fixations inside the AoI was different between the cases and CAVF (Table 1).

In the subgroup CAVF, the total duration of whole fixations was longer than in the controls (Table 1).

The analysis of the saccadic movements showed that the CAVF performed a greater number of saccades than controls (Table 1).

In summary in IReST©, our results indicated that these patients require more time to complete it, because of the greater number of fixations considering an AoI and their fixations are longer. In addition, these individuals had a greater number of saccades in the AoI.

In the DEM©, no differences were found in the numbers per minute between cases and controls (Table 2). It should be noted that the p values are at the limit of significance when the number of fixations in AoI and the mean duration of complete fixations were evaluated in CAVF (Table 2). Regarding the other variables, no significant differences were observed.

**Table 2.** Comparison between control group and cases in DEM test.

Parameter	Control group (*n*=30)	Cases (*n*=45)	Control (*n*=30) vs. Cases (*n*=45)	CNVF (*n*=33)	Control (*n*=30) vs. CNVF (*n*=33)	CAVF (*n*=12)	Control (*n*=30) vs. CAVF (*n*=12)
DEM	Mean *(SD)*	Mean *(SD)*	*p*	Mean *(SD)*	*p*	Mean *(SD)*	*p*
Numbers per minute	79.40 (1.81)	79.44 (1.41)	0.92	79.45 (1.39)	0.90	79.42 (1.51)	0.98
Number of whole fixations in AoI	104.80 (22.09)	113.69 (45.16)	0.42	109.42 (48.23)	0.94	125.42 (34.41)	0.05
Total duration of whole fixations (ms)	42796.07 (20731.28)	40124.62 (12678.78)	0.79	37961.91 (12523.59)	0.66	46072.08 (11581.15)	0.11
Average duration of whole fixations (ms)	324.37 (70.43)	352.60 (84.89)	0.14	345.79 (89.71)	0.30	371.33 (69.91)	0.06
Number of saccades in AoI	98.17 (26.89)	90.13 (27.41)	0.66	87.70 (28.64)	0.40	96.83 (23.49)	0.58

Note. vs.= versus, DEM = the test used is based on DEM*©* test, *SD*= Standard deviation, ms = millisecond, AoI = Area of Interest, CNVF = Cases with Normal Visual Field, CAVF = Cases with Affected Visual Field.

## Discussion

At the present, there is great interest in describing the relationship between central cognitive processes, such as attention, memory, and information processing and oculomotor alterations in neurodegenerative diseases (47).

In MS, fatigue is a frequent complaint. Recently, an association between fatigue and cognitive measures of attention has been identified. Patients with fatigue showed highly significant changes in their saccade dynamics, with a slowing of saccades. In addition, performance was influenced by disability and affective state. Therefore, other authors have concluded that, when controlling for disability and depression, saccadic stress tests could be used as an objective read-out for fatigue in MS patients (47).

Furthermore, in Parkinson ´s disease attention may be decreased and in conjunction saccades slowed (6; 8; 26; 48, 46, 47).

Meanwhile, the relationship between brain damage and reading difficulties in previously literate people without any associated impairment of spoken language or spelling and writing has been previously described (14). In that study, the investigators described two protocols for testing and training reading-related eye movements in adults. Unfortunately, those protocols do not seem to have been widely implemented.

The investigators used the OBER2 specialized computer-based, binocular, two-dimensional, infrared eye movement recording system (IOTA Eye Trace Systems, Sundsvall, Sweden) and the Visagraph II computer-based horizontal, infrared eye movement recording system for reading assessment (Taylor Associates, Huntington, Nueva York, United States of America). Nevertheless, the authors described that more than 1.5 hours were needed to compare the computer programs and apparatus for stimulus presentation and administration of the subjective reading rating scale questionnaire (14). They considered this time too long for using it with acquired brain disease patients. In this sense, eye-trackers are faster to use (21).

But no references have been found suggesting that patients with brain damage and VFD should be eliminated from evaluations with eye trackers because they can be a confounding factor. In this sense, we believe that this approach is original.

Previously, fixations and saccades during a text reading task were characterized with other eye tracking systems (EyeLink 1000) (35). In this work, the device Tobii Pro Nano® was evaluated, but due to the wide availability of different eye tracking models it would be very interesting to investigate the stability of the characteristics values and the results of the reliability evaluation of eye movement recordings (35).

The main limitation of the current research was the relatively small sample size, considering that several types of acquired brain diseases with different evolution times and different stages were included.

In addition, the sample's age range was extensive. In this regard, the age of patients should also be carefully considered in future studies because the evidence suggests that, elderly readers have longer fixations and shorter saccades, but skip more words and return to them more frequently than younger readers (18; 31; 33, 32).

This may explain why the differences in some parameters do not reach statistical significance when the subgroups were analyzed. Nevertheless, we believe that the current findings deserve further attention.

This study showed that simply quantifying the words per minute and numbers per minute during reading tests identified differences between the groups, but these differences were not significant. This can be explained by the great variability and dispersion of the results in the group of cases.

However, the use of the eye tracking system made it possible to establish differences in the characteristics of patients with brain damage and visual field involvement and of course provide information about their reading patterns.

The parameters considered most relevant were: quantitative and qualitative characteristics of the fixations and the number of complete saccades performed in general and considering an AoI.

It has been described that changes in peripheral vision, in patients with homonymous field defects, of the hemianopia or quadrantanopia type present in 30% to 85% of patients with acquired brain injury, are associated with less activation of their attentional brain mechanisms (24). In addition, in these patients there is also a predisposition to scan visual scenes using more frequent fixations (24).

Regarding complaints during the reading, although there are authors who have suggested that longer fixation durations generate slower reading and that this causes asthenopic symptoms. It is still unclear whether the symptoms influence the reading process, i.e. lead to a longer fixation duration, or whether the longer fixation duration causes asthenopic discomfort (16).

In addition, increased reading times in these patients could be interpreted as processing difficulties. In this sense, some authors refer that this may be a consequence of a greater number of regressions between words as an increase in the first step reading times resulting from the sum of all the fixations made in a region before a taken to another region (30; 44).

As a consequence, alterations in the registration of eye movements during reading may result from the reorganization of cognitive processes or the development of compensatory strategies not normally used for reading. We think it's interesting to be able to objectively identify these processes and to characterize them (9).

On the other hand, some authors have indicated that reading performance in patients with hemianopia depends on adaptive strategies such as predictive saccadic movements or eccentric fixation. The latter causes a displacement of the edge of the field towards the hemianopic side in conventional perimetry (42).

This results in an altered visual field shift to the hemianopic side in conventional perimetry and in enlarging the functional visual field (42).

Note that during eccentric fixation not only the sensory reference (the center of the visual field) must be changed, the oculomotor reference must also be changed (the center of the eye movement coordinates). This is also important for future rehabilitation approaches.

Eccentric fixation and frequent saccadic movements to the hemianopic side cause a visual field edge shift on conventional perimetry, which can be misinterpreted as macular sparing or even visual field improvement (42).

We consider that the objective study of oculomotor disturbances including measures of fixation, smooth tracking, and saccadic eye movement abnormalities should be included in future neurodegenerative disease trials. Therefore, the findings of this study should be taken into account when considering studying patients with VFD (27).

Although this study was a preliminary approach to use of this technology, it seems clear that in future work, larger homogeneous groups of patients with acquired brain diseases must be included.

It should be emphasized that there were no major differences between the cases and controls in the other parameters that measure the characteristics of the duration of whole fixations; this may be due to the large standard deviation.

In the DEM© test, the number of whole fixations varied with respect to the text test, possibly because reading numbers requires less comprehension than reading a text.

In summary, the use of the eye tracker identified several altered parameters in the reading pattern, mainly in patients with brain damage and VFD. Therefore, it is important to consider VFDs before evaluating patients with eye-tracker systems. Also, we encourage the use of objective methods and standardized procedures for evaluating gaze data in reading anomalies.

Future studies that focus on groups of specific pathologies should obtain information that facilitates a better understanding of patients' reading problems.

### Ethics and Conflict of Interest

The authors declare that the contents of the article are in agreement with the ethics described in http://biblio.unibe.ch/portale/elibrary/BOP/jemr/ethics.html and that there is no conflict of interest regarding the publication of this paper.

### Acknowledgements

This study was supported by grants from the Fundación Eugenio Rodríguez Pascual, Call 2021, Spain, and the Gerencia Regional de Salud de Castilla y León (grant no. GRS 2498/A/22). Agustín Mayo-Iscar research was partially supported by grant PID2021-128314NB-I00 funded by MCIN/AEI/10.13039/501100011033/FEDER.
